# Supplementary material for: A sexually dimorphic pre-stressed translational signature in CA3 pyramidal neurons of BDNF Val66Met mice
Source: Nat Commun. 2017 Oct 9;8:808. doi: 10.1038/s41467-017-01014-4 (PMC5634406; doi:10.1038/s41467-017-01014-4)
Supplement: Supplementary file 1 — Supplementary Information [file 41467_2017_1014_MOESM1_ESM.pdf]

Supplementary Figure 1

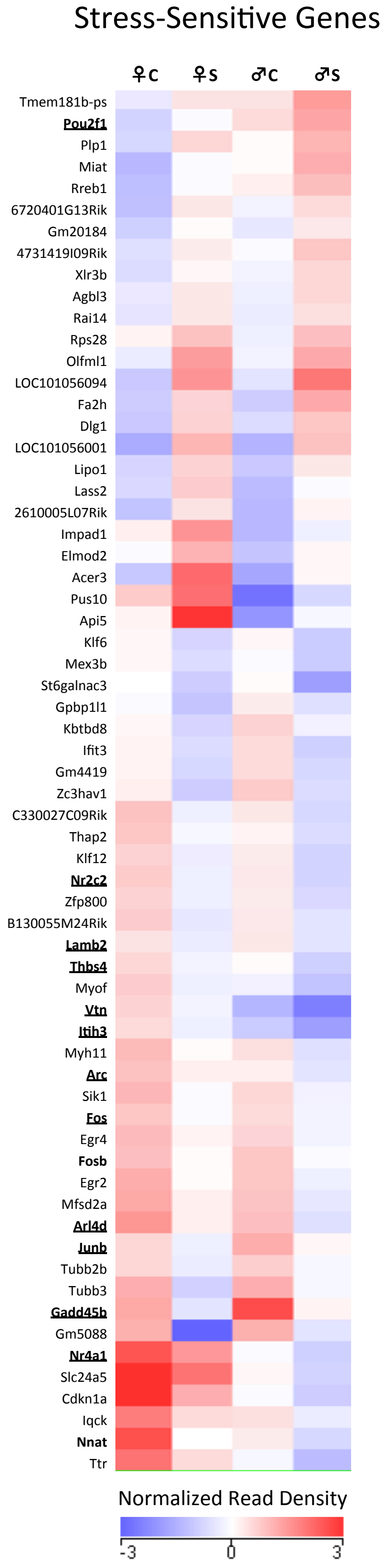

Commonality in stress-dependent gene expression change in males and females. Heat map representing the normalized read density of the 65 genes that are either up-regulated (FC>=1.5, p<0.001) or down-regulated (FC<=-1.5, p<0.001) after stress in both male and female BDNF$^{+/+}$ mice. Genes in bold have a known function in stress. C: unstressed BDNF$^{+/+}$; S: stressed BDNF$^{+/+}$.

Supplementary Figure 2

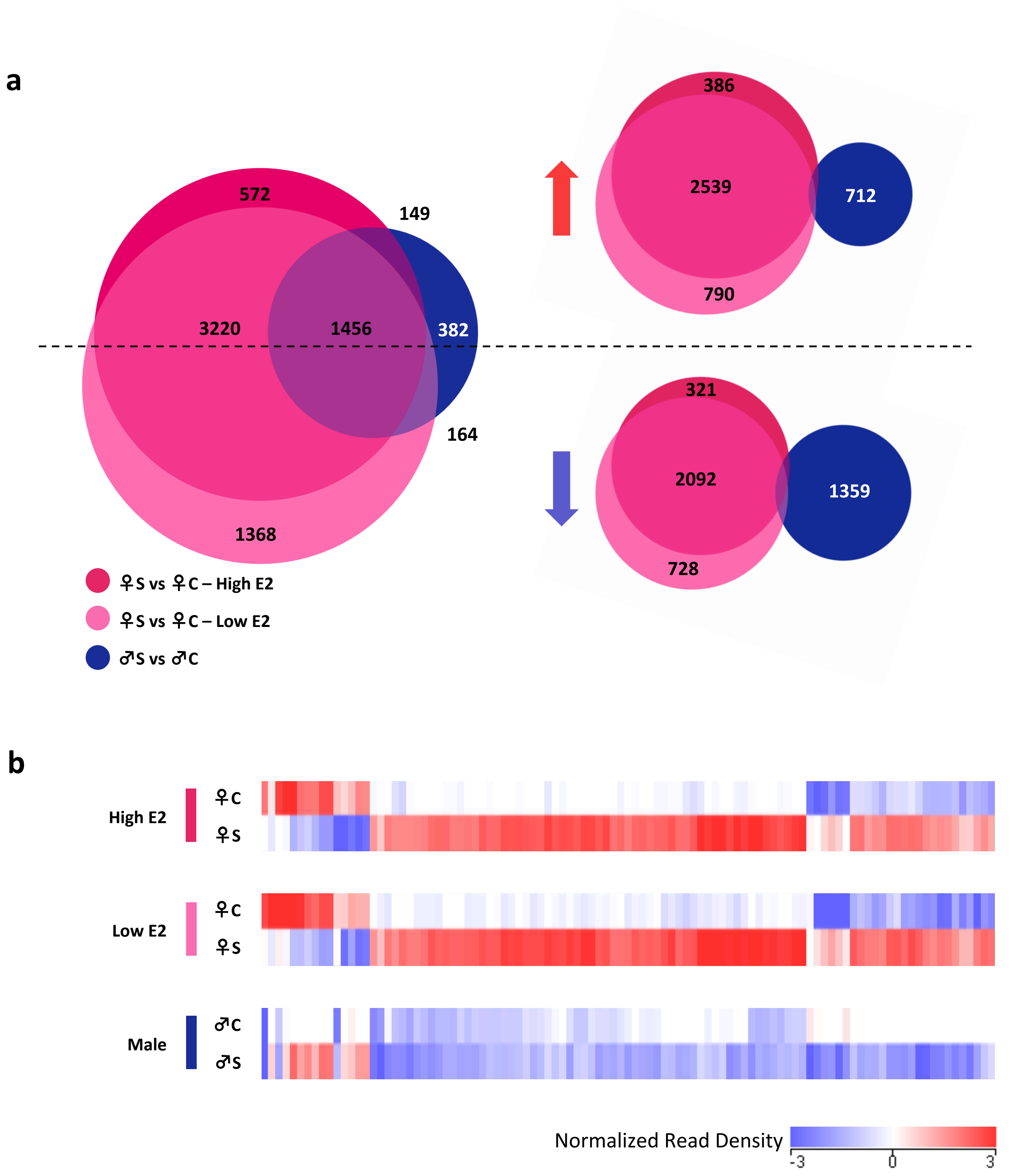

Translational profile in response to stress of CA3 pyramidal neurons is similar across oestrous cycle stages. (a) (Left) Venn diagram depicting the number of genes altered by acute stress in females in proestrus (high oestradiol, dark pink circle), females in metoestrus/dioestrus (low oestradiol, light pink circle), and males (blue circle) (Z-score < 0.001; absolute fold change > 1.5). (Right) Venn diagrams are broken into up-regulated (red arrow) and down-regulated (blue arrow) genes. (b) Heat map representing the normalized read density of the 100 genes with the highest variance across all groups. C: unstressed BDNF$^{+/+}$; S: stressed BDNF$^{+/+}$; E2: estradiol.

Supplementary Figure 3

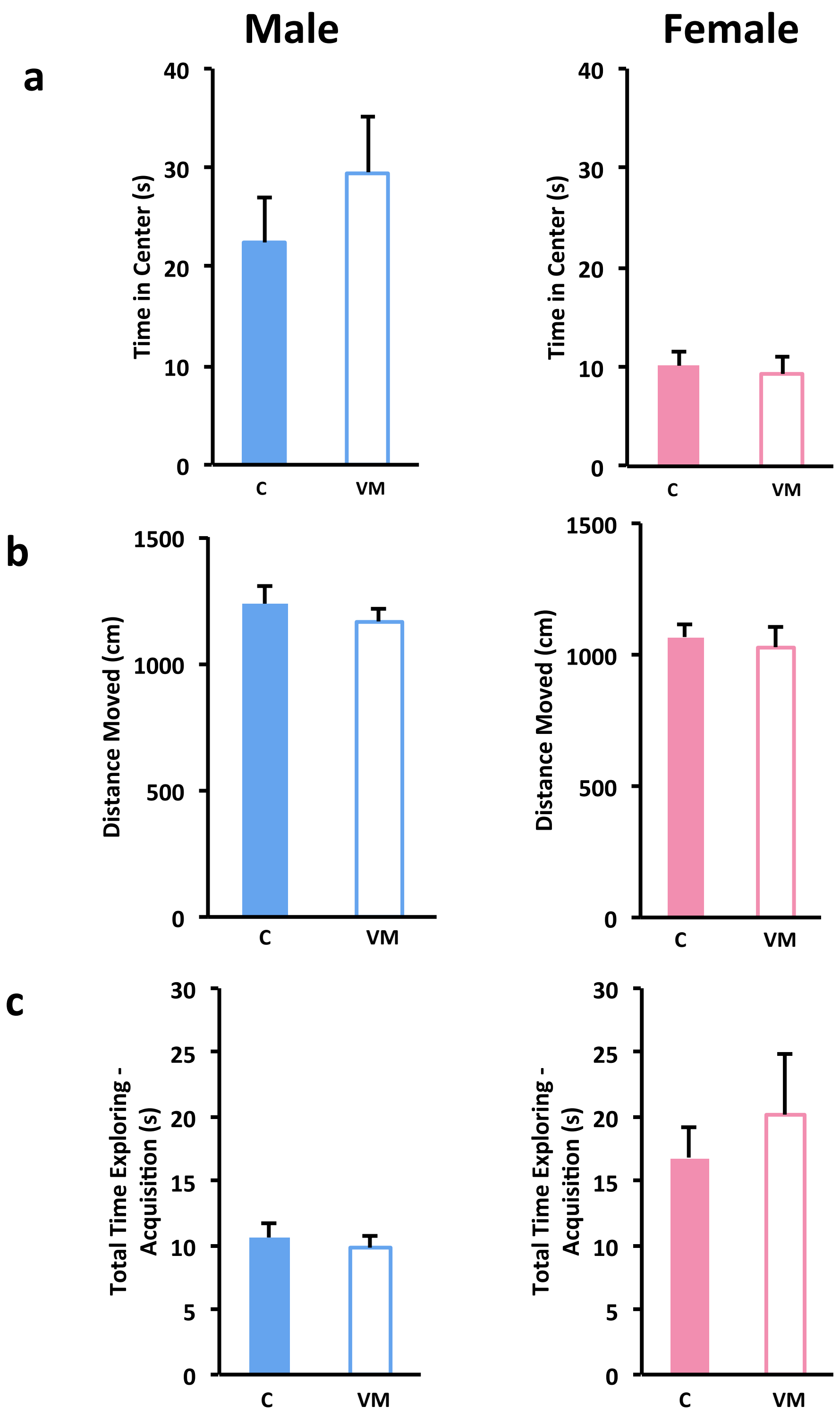

Behavioural similarities between sexes and genotypes. (a) Time spent in the centre of the arena and (b) distance travelled in the open field during the habituation phase. (c) Total time exploring either object during the acquisition phase. Values are mean $\pm$ SEM of 19-29 determinations. T-tests are negative for all of the comparisons. *p<0.05* C: unstressed $BDNF^{+/+}$ (n=19 males, n=28 females); VM: unstressed $BDNF^{Met/+}$ (n=29 males, n=20 females).

Supplementary Figure 4

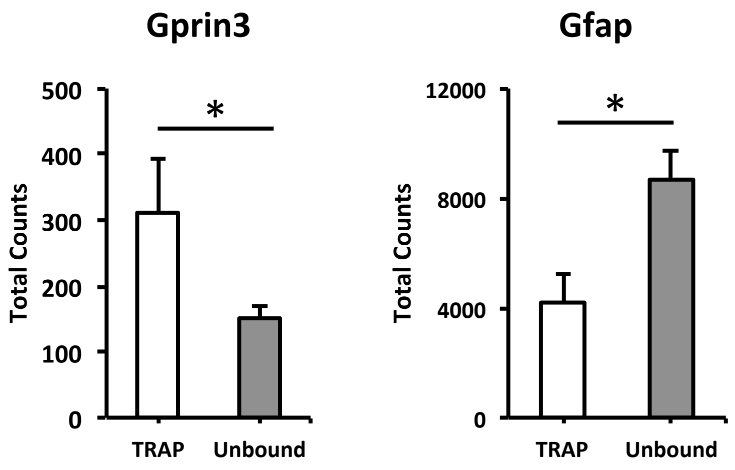

Gprin3 is enriched in TRAP fraction. Raw counts of translating ribosome affinity purification-immunoprecipitation (TRAP-IP) and unbound mRNA isolations for *Gprin3* in the TRAP fraction and the glial specific marker *Gfap* in the unbound fraction. Values represent the mean $\pm$ SEM of 8 determinations (combination of both sexes, genotypes and stress treatments) per group. *z-score ≤ 0.001.
